# Novel Dual Mechanism GRT‑X Agonist Acting on Kv7 Potassium Channel/Translocator Protein Receptor Prevents Motoneuron Degeneration Following Exposure to Mouse and Human Amyotrophic Lateral Sclerosis/Frontotemporal Dementia Astrocyte-Conditioned Media

**DOI:** 10.1021/acschemneuro.5c00197

**Published:** 2025-07-17

**Authors:** Vera M. Masegosa, Elsa Fritz, Daniela Corvalan, Fabiola Rojas, Polett Garcés, Xavier Navarro, Petra Bloms-Funke, Brigitte van Zundert, Mireia Herrando-Grabulosa

**Affiliations:** † Department of Cell Biology, Physiology and Immunology, Institute of Neurosciences, 16719 Universitat Autònoma de Barcelona, Bellaterra 08193, Spain; ‡ Centro de Investigación Biomédica en Red (CIBER), Instituto de Salud Carlos III, Madrid 28029, Spain; § Institute of Biomedical Sciences (ICB), Faculty of Medicine & Faculty of Life Sciences, 28087 Universidad Andres Bello, Santiago 8370035, Chile; ∥ 14938 Grünenthal GmbH, Aachen 52099, Germany; ⊥ Department of Neurology, University of Massachusetts Chan Medical School (UMMS), Worcester, Massachusetts 01655, United States; # Millennium Nucleus of Neuroepigenetics and Plasticity (EpiNeuro), Santiago 8370035, Chile

**Keywords:** GRT-X, astrocyte conditioned medium, amyotrophic lateral sclerosis, frontotemporal dementia, motoneuron death, oxidative and excitotoxity stress

## Abstract

Amyotrophic lateral sclerosis (ALS) and frontotemporal dementia (FTD) form a continuous spectrum of aggressive neurodegenerative diseases affecting primarily motoneurons (MNs) and cortical frontotemporal neurons. Noncell autonomous mechanisms contribute to ALS/FTD, wherein astrocytes release toxic factor(s) detrimental to MNs. Because of the multifactorial nature of ALS, single-pathway-focused therapies have limited effectiveness in improving ALS. Therefore, novel combinatorial therapies are currently being pursued. Here, we evaluated whether the simultaneous activation of two complementary targets, the voltage-gated potassium channels 7.2/3 (Kv7.2/3) and the mitochondrial translocator protein (TSPO), by a novel synthesized compound (GRT-X) is an effective neuroprotective treatment in ALS in vitro models. We exposed primary rat ventral spinal cord neuronal cultures and rat spinal cord organotypic cultures to astrocyte-conditioned medium derived from primary mouse ALS astrocytes expressing mutant human SOD1 (SOD1^G93A^-ACM) or from human-induced pluripotent stem cell (iPSC)-derived astrocytes carrying an ALS-causing mutation in SOD1 (SOD1^D90A^-ACM) or an ALS/FTD-causing mutation in TDP-43 (TDP43^A90 V^-ACM). We report that the diverse human and mouse ALS/FTD-ACMs compromise the MN viability. Remarkably, GRT-X led to consistent protection of MNs. Moreover, ALS/FTD-ACM increases oxidative stress levels, which are prevented with GRT-X treatment. Together, we show that the complementary activation of TSPO and Kv7.2/3 may offer a novel therapeutic strategy for ALS/FTD due to its capacity to protect MNs from noncell-autonomous toxicity induced by diseased astrocytes.

## Graphical abstract

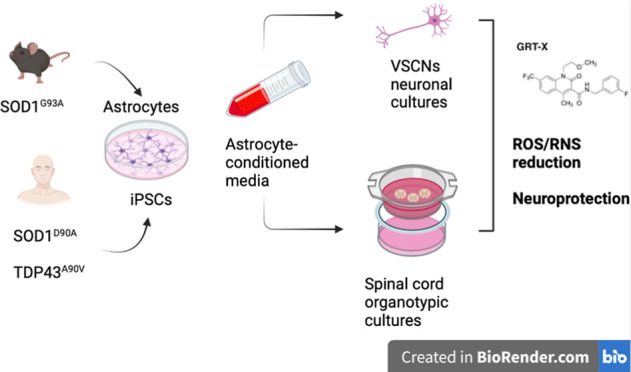

## Introduction

Motoneuron (MN) diseases are a heterogeneous group of diseases that cause progressive degeneration of the MNs. The most common type is amyotrophic lateral sclerosis (ALS), a devastating neurodegenerative disease characterized by the selective degeneration of both upper and lower MNs, resulting in death, in most cases, by respiratory failure within 3–5 years after diagnosis. Around 90–95% of the cases are sporadic with no apparent genetic link (sporadic ALS, sALS), while the remaining 5–10% of the cases are related to several genetic mutations (familiar ALS, fALS).^1^ Since the first gene was discovered in fALS, superoxide dismutase 1 (SOD1) more than 200 genetic variants of this gene, has been reported (https://alsod.ac.uk/output/variant.php/35). SOD1^G93A^ variant led to the development of the first mouse model for ALS where mutant human SOD1^G93A^ is ubiquitously overexpressed.^2^ Despite 3 decades of investigation, the primary molecular mechanisms by which SOD1 mutations cause MN disease remain unclear, with a plethora of toxic mechanisms proposed (see below), likely through the generation of misfolded SOD1, independent of alterations in its dismutase activity.^3^

Among other ALS-causing genes, mutations in the TARDBP gene, encoding for 43 kDa TAR DNA-binding protein (TDP-43), are related to autosomal dominant ALS and account for 1–2% of ALS cases.^4^ Patients carrying TARDBP gene mutations can develop ALS, frontotemporal dementia (FTD), or both and hence exhibit motor dysfunction and cognitive deficits.^5,6^ Although the precise molecular mechanisms remain undefined, TDP-43 pathology—whether caused by mutations or pathological phosphorylation, as seen in most ALS/FTD cases—is characterized by a combination of cytoplasmic aggregation and nuclear clearance.^3^ Accumulating evidence indicates that nuclear depletion of TDP-43 disrupts RNA metabolism, particularly by impairing the repression of nonconserved cryptic exon splicing of specific genes such as UNC13A and Stathmin-2.^7^

Many pathogenic mechanisms contribute to MN degeneration in ALS, such as oxidative stress, glutamate excitotoxicity, endoplasmic reticulum stress, protein misfolding, polyphosphate-mediated hyperexcitability, and others.^1,8,12,13^ Moreover, the contribution of non-neuronal cells to ALS progression was demonstrated in animals since wild-type MNs developed ALS signs when surrounded by ALS mutant gene-expressing non-neuronal cells such as astrocytes.^9^ Cultures of wild-type MNs with mouse or human astrocytes harboring ALS/FTD mutant genes also evidenced the astrocytic noncell autonomous effect in MN degeneration.^9–12^ Thus, primary ALS and ALS/FTD astrocytes derived from transgenic mouse models harboring pathogenic gene mutations in SOD1, TARDBP, and C9ORF72 reduce healthy wild-type MNs in cocultures or after application of astrocyte-conditioned medium (ACM).^13–18^ Chronic infusion of ACM obtained from astrocyte harboring the SOD1^G93A^ mutation triggers spinal MN death and neuromuscular dysfunction in healthy rats.^19^ Decreased MN survival was also found in studies with human-induced pluripotent stem cell (iPSC)-astrocytes (or derived ACM) carrying mutations in SOD1,^20,21^ C9ORF72,^21,22^ or TARDBP^12,13^ genes. These studies have shown that independent of the species (mouse versus human) and mutant gene (SOD1, TARDBP, and C9ORF72), ALS astrocytes excessively release soluble factors, including glutamate, reactive oxygen and nitrogen species (ROS/RNS), ATP, various cytokines and chemokines, and inorganic polyphosphates (polyP). PolyP, a ubiquitous and negatively charged biopolymer, is enriched and excessively released by both mouse and human iPSC-derived astrocytes harboring pathogenic mutations in SOD1, TARDBP, and C9ORF72.^12,13^ Through gain- and loss-of-function experiments, it was also demonstrated that this aberrant release of polyP from ALS and ALS/FTD astrocytes is neurotoxic, promoting MN death by inducing neuronal hyperexcitability and consequent Ca^2+^ overload.^12,13^

Currently, there is no effective therapy for ALS; varying on different countries, only anti-excitotoxic riluzole, antioxidant edaravone, and the newest therapy based on reductions in plasma neurofilament light chain, tofersen, are currently available; therefore, finding novel and combinational therapeutic strategies is currently being pursued. Here, we used GRT-X (N-[(3-fluorophenyl)-methyl]-1-(2-methoxyethyl)-4-methyl-2-oxo-(7-trifluoromethyl)-1H-quinoline-3-carboxylic acid amide), a small molecule that simultaneously targets the mitochondrial translocator protein (TSPO) and the voltage-gated potassium channels 7.2/3 (Kv7.2/3).^23^ It has been recently demonstrated that GRT-X has a neuroprotective and neuroregenerative effect following lesion of cervical spinal nerves in rats.^23^ Furthermore, GRT-X was effective in reducing seizures in rodent models of epilepsy.^24^

TSPO is expressed in glial cells, neurons, and endothelial and ependymal cells and localized in the outer mitochondrial membrane in enriched steroidogenic regions.^25^ Its function has been widely associated with steroidogenesis,^26^ but also linked to other functions such as mitochondrial bioenergetics,^27,28^ redox mechanisms,^29,30^ and neuroinflammation.^31^ Enhanced TSPO levels in glial cells and neurons were reported in pathological conditions, including ALS, whereas under physiological conditions, TSPO is poorly expressed.^32^ Thus, TSPO is an interesting target for ALS.

Olesoxime (also known as TRO19622), a ligand of TSPO, caused a concentration-dependent increase in MN survival, reduced oxidative stress levels, and promoted neurite outgrowth in in vitro studies.^33^ In SOD1^G93A^ mice, olesoxime improved the decline of performance on the grid test and rotarod and increased survival.^33^ Reduced microglial and astroglial activation and attenuated MN loss and muscle denervation were observed in olesoxime-treated ALS mice.^34^ However, when tested as an add-on therapy in a phase II–III clinical trial in people with ALS, olesoxime did not increase the low clinical benefit of riluzole,^35^ which may reflect the challenges in designing and conducting ALS clinical trials.^36^ Furthermore, the results may suggest that these preclinical models may not always be translationally predictive of the clinical efficacy.

Heteromeric Kv7.2/3 channels underlie the M-current, which stabilizes the resting membrane potential and reduces neuronal excitability.^37^ Since hyperexcitability is a common feature of ALS,^38^ potassium channels are an attractive target for ameliorating the disease. Retigabine was shown to reduce hyperexcitability, ROS production, and MN bursting and prevent MN loss in rat hypoglossal MN cultures.^39^ Retigabine (also known as ezogabine), an approved antiepileptic drug, also blocked hyperexcitability and increased in vitro survival of SOD1^A4 V/+^ ALS hiPSC-derived MNs,^40^ and a phase II randomized clinical trial demonstrated its effectiveness in reducing MN excitability in people with ALS.^41^ ICA-27243, a highly selective Kv7.2/3 opener, has been found to prevent spinal MN loss in spinal cord explants under excitotoxic conditions; in female SOD1^G93A^ mice in vivo, ICA-27243 preserved neuromuscular function and enhanced motor activity.^42^ These data highlight that Kv channels, especially Kv7.2/3, represent a potential target for mitigating hyperexcitability in a range of diseases, including ALS.

The pharmacological responses produced by compounds that activate either the Kv7.2/3 potassium channel or mitochondrial protein TSPO should be retained by a compound capable of activating both targets. The small dual mode-of-action molecule GRT-X (N-[(3-fluorophenyl)-methyl]-1-(2-methoxyethyl)-4-methyl-2-oxo-(7-trifluoromethyl)-1H-quinoline-3-carboxylic acid amide, see Figure 2A)^43,44^ can simultaneously activate Kv7.2/3 and TSPO, and is, therefore, expected to maintain the pharmacological response of the individual compounds and act as a neuroprotective agent. As shown in our previous studies,^23^ GRT-X activated neuronal Kv7.2/3 channels and induced a hyperpolarization of the membrane resting potential with high potency and relative efficacy, suggesting GRT-X potential to prevent hyperexcitability and, thereby, offer neuroprotection. The binding affinity of GRT-X to rat TSPO was high, and while its steroidogenic efficacy in vitro in rat C6 glioma cells was moderate, in vivo it was significant, with antihyperalgesic, anticonvulsant, and neuroprotective effects in rats. In a model of severe crush injury of the cervical spinal nerves in rats in vivo, GRT-X promoted survival, regrowth, and functional recovery of spinal MNs.

Given the neuroprotective effects of GRT-X and its targets’ relevance in ALS therapy, this study aimed to provide evidence of the neuroprotective potential of GRT-X in a battery of ALS/FTD in vitro models. We propose that a multitargeted treatment that acts beneficially and simultaneously on the mitochondrial protein TSPO and the Kv7.2/3 potassium channel promises a more effective approach to alleviating ALS/FTD. In this study, we assessed if GRT-X, which has been demonstrated simultaneously to activate TSPO and Kv7.2/3 ^23^, may prevent MN loss and ROS production in dissociated rat primary spinal cord MN cultures (VSCNs) and rat SCOC treated with ACM collected from human and mouse astrocytes with ALS/FTD-linked mutations in SOD1 and TARDBP genes.

## Results and Discussion

Single activation of the Kv7.2/3 potassium channel by ICA-27243 ^42^ and of the mitochondrial protein TSPO by olesoxime exerted a neuroprotective effect on MNs in spinal cord organotypic cultures (SCOCs) under glutamate-induced excitotoxic conditions. The efficacy and potency of olesoxime in the low micromolar concentration range confirm previous results showing neuroprotection of primary MN cell cultures.^33^

We hypothesized that combining the two compounds ICA-27243 and olesoxime, which involve diverse targets and multiple signaling pathways, may promote the complex processes of neuroprotection and hence lead to superior efficacy than the individual compounds.

### Synergistic Effect of ICA-27243 and Olesoxime on MN Preservation in SCOCs

Combination impact of ICA-27243 and olesoxime on MN viability was evaluated within glutamate exposed SCOCs. Spinal cord explants were exposed to l-glutamic acid (50 μM) for 30 min at 15 DIV, together with treatments with the test compounds (Figure 1A). The neuroprotective effect of ICA-27243 in glutamate exposed SCOCs has been recently reported, showing that application of 10 μM ICA-27243 significantly increased MN survival rates to 82 ± 5% compared to 61 ± 2% MNs preserved after 50 μM glutamate, while other test concentrations of ICA-27243 were ineffective (65 ± 2% after 2.5 μM, 68 ± 2% after 5 μM, and 67 ± 9% MN preserved after 20 μM, respectively).^42^ Here, we first tested olesoxime at different concentrations (2.5 μM, 5 μM, 10 μM, and 20 μM) in SCOCs subjected to excitotoxicity (Figure 1B,C). In line with previous studies, the number of MNs preserved, evaluated as SMI32+ cells within the ventral horn of L4-L5 slices, was significantly reduced by adding 50 μM glutamate (60 ± 2% MN preserved) compared to control slices (100 ± 2% MN preserved). Olesoxime treatment at 5, 10, and 20 μM showed significant reductions in MN death (72 ± 5; 90 ± 5; 75 ± 5% MN preserved, respectively). A concentration of 2.5 μM was ineffective in preserving MN (53 ± 2% MN preserved) (Figure 1B,C).

**Figure 1:**
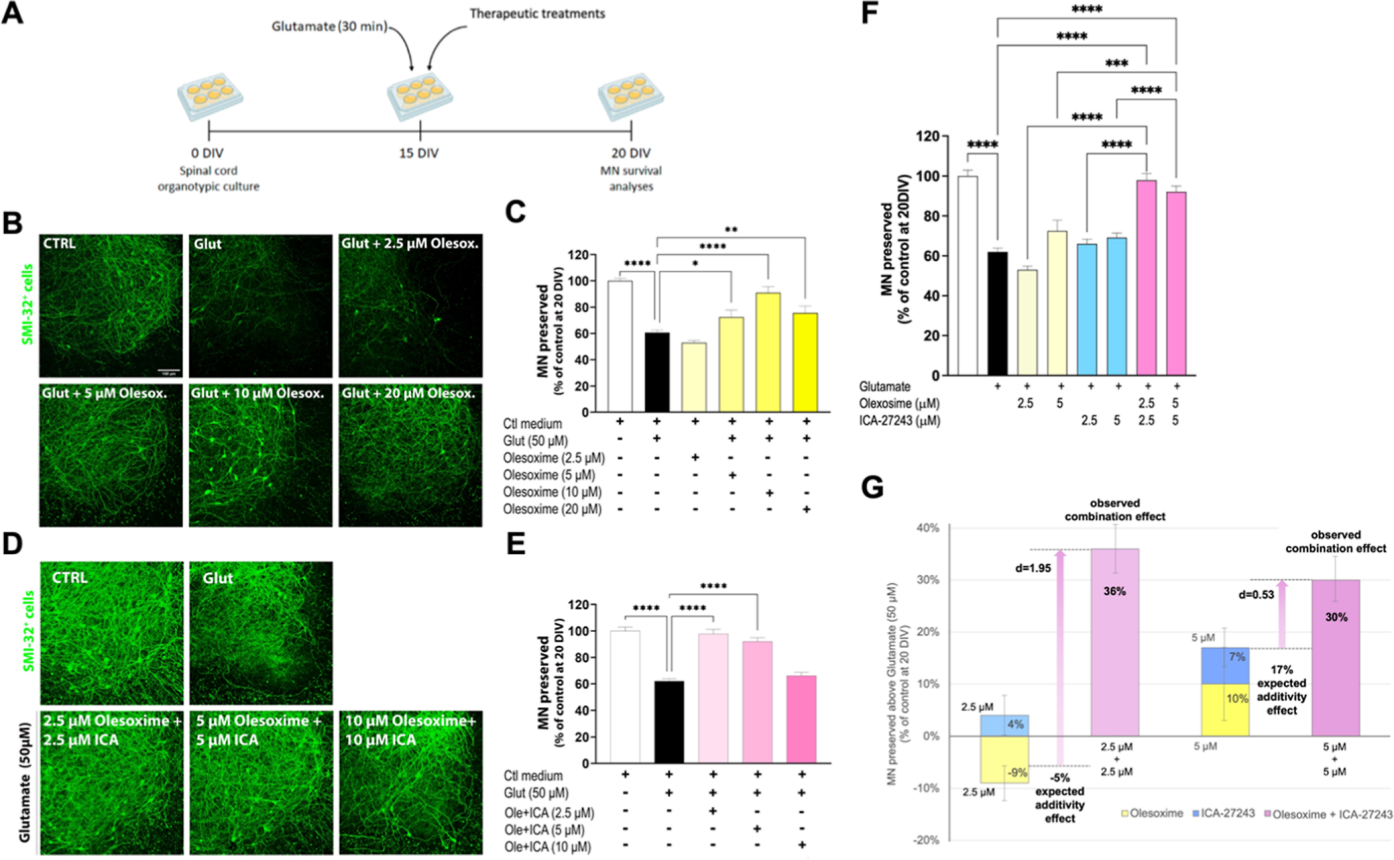
Unimodal combinational treatment with ICA-27243 and olesoxime. (A) Experimental design of SCOC under glutamate excitotoxicity. (B) Representative confocal images of the ventral horn of spinal cord hemislices immunostained with SMI32 at 20 DIV of SCOCs exposed to glutamate and treated with olesoxime. Scale bar 100 μm. (C) Bar graph showing the neuroprotective effect of 5 μM, 10 μM, and 20 μM of olesoxime in SCOCs exposed to glutamate. Data are shown as mean ± SEM (*n* = 15–34 hemisections per treatment). One-way ANOVA followed by Bonferroni’s post hoc test: ****p < 0.0001; **p < 0.01; *p < 0.05 versus glutamate condition. (D) Representative confocal images of the ventral horn of spinal cord hemislices immunostained with SMI32 at 20 DIV of SCOCs exposed to glutamate and treated with the combination of ICA27243 and olesoxime. Scale bar 100 μm. (E) Bar graph showing the neuroprotective effect of 2.5 μM and 5 μM of each compound in SCOCs exposed to glutamate. Data are shown as mean ± SEM (*n* = 11–19 hemisections per treatment). One-way ANOVA followed by Bonferroni’s post hoc test: ****p < 0.0001 versus glutamate condition. (F) Bar graph of the interactions between olesoxime and ICA-27243. Observed combination effects after treatment with equimolar combinations of olesoxime and ICA-27243 (2.5 μM + 2.5 μM and 5 μM + 5 μM, respectively) are compared with that after treatments with either olesoxime or ICA-27243 by one-way ANOVA followed by Tukey’s test (****p < 0.001; *n* = 20–30) and (G) with the expected additivity effects. Equimolar combination of olesoxime and ICA-27243 at 2.5 μM (98 ± 3% MNs preserved) and 5 μM (92 ± 3% MNs preserved) shows synergism effect compared with olesoxime treatment at 2.5 μM and 5 μM (53 ± 3%; 72 ± 5%, respectively) and ICA-27243 treatment at 2.5 μM and 5 μM (66 ± 2%; 69 ± 2%, respectively) under glutamate condition (62 ± 2%). Data are shown as mean ± SEM. Effect sizes are indicated as Cohen’s *d* values.

Once the protective concentration ranges of the individual compounds were established, treatments with equimolar combinations of olesoxime and ICA-27243 were tested to evaluate a synergistic-type of effect. Again, the addition of glutamate significantly reduced the number of MNs by 38% (62 ± 2% MN preserved) compared to the control slices (100 ± 3% MN preserved). Treatment with the combination of olesoxime and ICA-27243 significantly preserved MNs, with concentrations of 2.5 μM each and 5 μM each proving effective (98 ± 3; 92 ± 3% MN preserved), while the combination of 10 μM of each compound was ineffective (66 ± 3% MN preserved) (Figure 1D,E). Importantly, a full rescue of the MNs was demonstrated after combined treatment with a concentration of 2.5 μM of each compound, which was a subthreshold for each individual compound. Furthermore, the observed combination effect after treatment with 5 μM of each compound was above the expected additivity effect. In order to have a better indication of the magnitude of synergistic interaction between olesoxime and ICA-27243, we calculated the effect size of the observed versus expected additivity effects after treatment with 2.5 and 5 μM of each compound. Cohen’s d values were 3.28 and 0.93 after equimolar combinations of (2.5 μM + 2.5 μM) and (5 μM + 5 μM), respectively, indicating a large effect size for both combinations. In summary, the data show synergistic neuroprotective effects of olesoxime and ICA-27243 (Figure 1F,G).

In fact, the present study showed supraadditive protection of MNs after combined treatment with ICA-27243 and olesoxime at equimolar concentrations of 2.5 and 5 μM of each compound in SCOCs under excitotoxic conditions. The analysis of the effect size confirmed that the observed effects were larger than the expected additivity effects, indicating a large synergistic effect. Further investigations on the synergistic-type of interaction between ICA-27243 and olesoxime are warranted, such as to explore the optimal combination ratio and the relevance of the various mechanisms, e.g., hyperpolarization,^31^ steroidogenesis,^26^ mitochondrial bioenergetics,^27,28^ redox mechanisms,^29,30^ and neuroinflammation.^31^

### Neuroprotective Responses by the Small Dual Mode-of-Action Molecule GRT-X under Excitotoxic Conditions

First, we investigated whether the small molecule GRT-X exerts neuroprotective effects in the SCOCs under excitotoxic conditions. Acute excitotoxic damage induced by adding glutamate at 50 μM for 30 min reduced the number of SMI-32^+^ MNs in the ventral horn by 45% (55 ± 2% MN preserved), compared to untreated slices (control, 100 ± 2% MN preserved; Figure 2B–D). GRT-X treatment at 6.25, 12.5, and 25 μM showed a significant concentration-dependent reduction in MN death (45 ± 4; 76 ± 3; 85 ± 3% MN preserved, respectively), reaching a maximum protective effect at 25 μM. No MN loss was detected in SCOCs under vehicle (DMSO) when exposed to Locke solution without glutamate. In SCOCs, GRT-X provided protection at 12.5 and 25 μM, consistent with the concentration range of 10–50 μM of GRT-X that increased pregnenolone synthesis in rat glioma C6 cells.^23^ Hence, these results suggest that TSPO-mediated steroidogenesis may contribute to neuroprotection by GRT-X in SCOCs, although further experiments will be needed to corroborate this mechanism. It should be mentioned that there is controversy regarding the involvement of TSPO in steroidogenesis. Despite reports that the knockout of TSPO did not induce changes in steroidogenesis,^45,46^ other studies showed that TSPO-KO mice have altered steroidogenic flux and reduced total steroidogenic output,^47^ and CRISPR/Cas9 TSPO deficiency reduced progesterone levels and steroid formation.^26^ In a previous study, GRT-X also stimulated biosynthetic pathways in adult rats in vivo and increased brain concentrations of pregnenolone, progesterone, deoxycorticosterone, corticosterone, and 3α,5α-reduced metabolites, but did not affect levels of testosterone.^23^ Noteworthy, increased neurosteroid levels are beneficial by attenuating excitotoxicity, neuroinflammation, oxidative stress, and neuronal degeneration.^48^ Additionally, it was also proven that GRT-X activated Kv7.2/3 channels with an EC_50_ of 0.37 ± 0.15 μM in patch-clamp recordings in recombinant CHO-K1 cells, which is clearly lower than the neuroprotective concentration range in SCOCs. A similar profile was found for ICA-27243, which was neuroprotective in SCOCs at a concentration of 10 μM,^42^ however, activated Kv7.2/3 channels with an EC_50_ = 0.68 ± 0.05 μM.^23^ One explanation could be that a higher degree than 50% of Kv7.2/3 channel activation may be required to prevent toxic hyperexcitability in SCOCs. A more detailed analysis in SCOCs will be required to better understand the significance of Kv7.2/3 activation on MN survival as well as the underlying mechanisms of neuroprotection after treatment with GRT-X under these experimental conditions.

**Figure 2:**
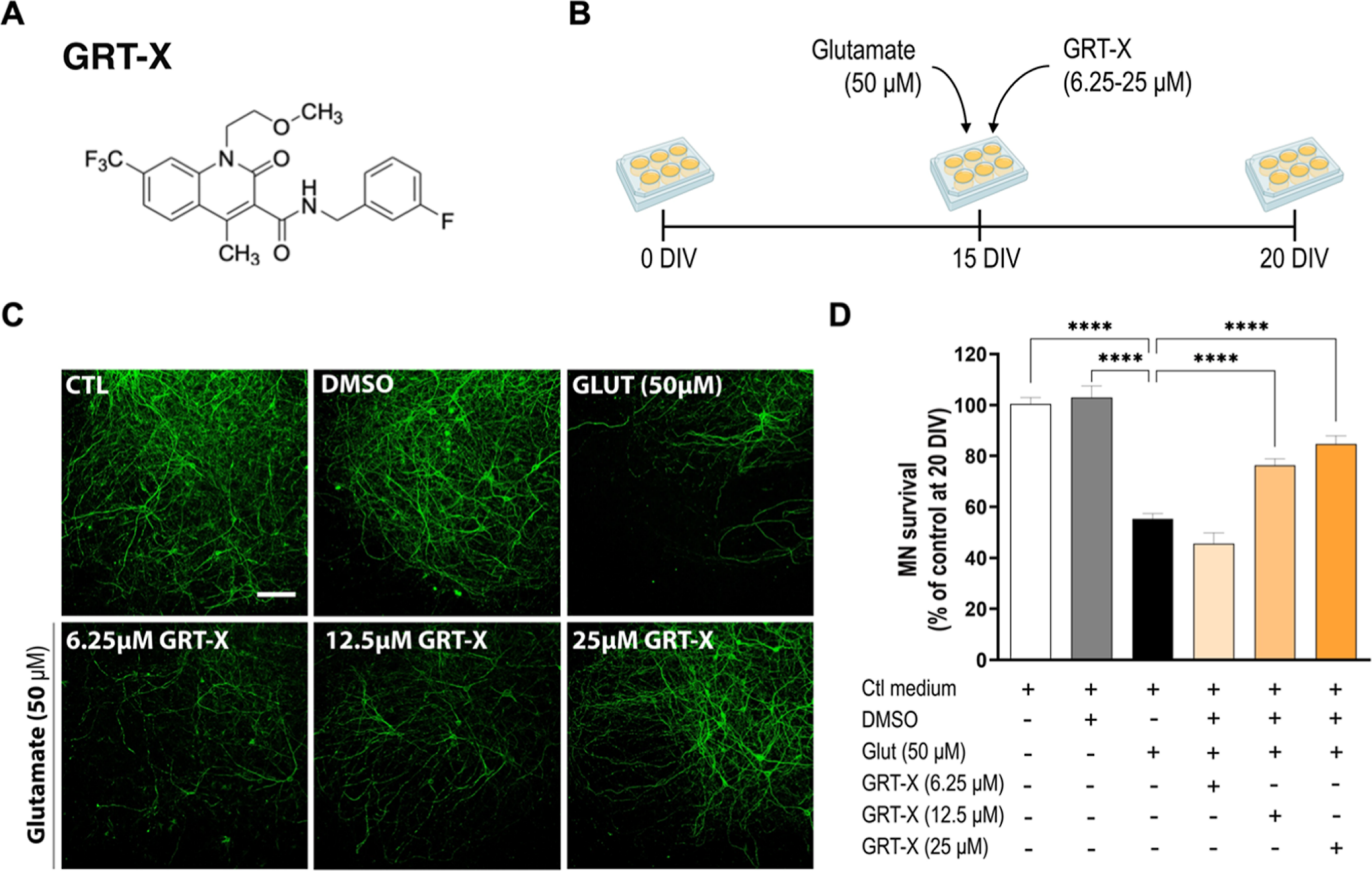
Neuroprotective effect of GRT-X under excitotoxic condition. (A) Chemical structure of GRT-X (N-[(3-fluorophenyl)-methyl]-1-(2-methoxyethyl)-4-methyl-2-oxo-(7-trifluoromethyl)-1H-quinoline-3-carboxylic acid amide). GRT-X synthesis procedure is description.^42,43^ (B) Experimental design of the evaluation of GRT-X neuroprotective capacity in spinal cord organotypic cultures (SCOCs) under excitotoxicity conditions (50 μM glutamate exposure for 30 min). (C) Representative images of 20 days in vitro (DIV) spinal cord ventral horns exposed to glutamate (Glut 50 μM) in the presence and absence of GRT-X at three concentrations (6.25, 12.5, and 25 μM). Motoneurons (MNs) were visualized in fixed slices with immunofluorescence staining for SMI32^+^ (green). Scale bar 100 μm. (D) Plot showing the percentage of SMI-32^+^ surviving MNs in the ventral horn of spinal cord hemislices after glutamate exposure with or without GRT-X treatment. Untreated slices (Ctl medium) and vehicle (DMSO) were used as controls. Data are shown as mean ± SEM. One-way ANOVA with Bonferroni’s post hoc test: ****p < 0.0001 versus glutamate condition or control condition).

### GRT-X Rescues MNs from Death in VSCNs and SCOCs when Exposed to Mouse SOD1^G93A^-ACM

In order to evaluate the neuroprotective potential of GRT-X in the context of ALS disease, it was assessed in in vitro models based on the VSCNs and SCOCs exposed to ALS/FTD-ACMs.

First, we investigated whether application of GRT-X influences MN survival in rat primary spinal cord cultures (VSCNs) under control conditions (i.e., without toxic agents). A concentration–response curve of GRT-X was performed in order to determine the maximum concentration of GRT-X tolerated by MNs in VSCNs when treated from 4 to 7 DIV (Figure 3A). At 7 DIV, VSCNs were fixed, and neuronal survival was assayed by double immunostaining for anti-MAP2 to identify all neurons and anti-SMI32 ^13,18^. In agreement with our previous study,^13^ these SMI32^+^/MAP2^+^ MNs, but not SMI32^–^/MAP2^+^ interneurons, fulfill two important criteria in confirming the identification of MNs: first, they have a typical MN morphology, containing a large soma (>20 μm) and extending at least five primary dendrites; second, they also stain positive for choline acetyltransferase (ChAT), an enzyme responsible for biosynthesis of the neurotransmitter acetylcholine expressed in MNs (Supporting Information, Figure 1). In VSCNs under control conditions, data show that concentrations up to 2.5 μM GRT-X did not significantly reduce MN survival (Figure 3B). Therefore, concentrations of 0.5 μM, 1 μM, and 2.5 μM of GRT-X were chosen for further studies performed in VSCNs. The neuroprotective doses in SCOCs were higher than the toxic doses observed in primary VSCNs, likely due to differences in cellular and culture medium compositions between these in vitro models.

**Figure 3:**
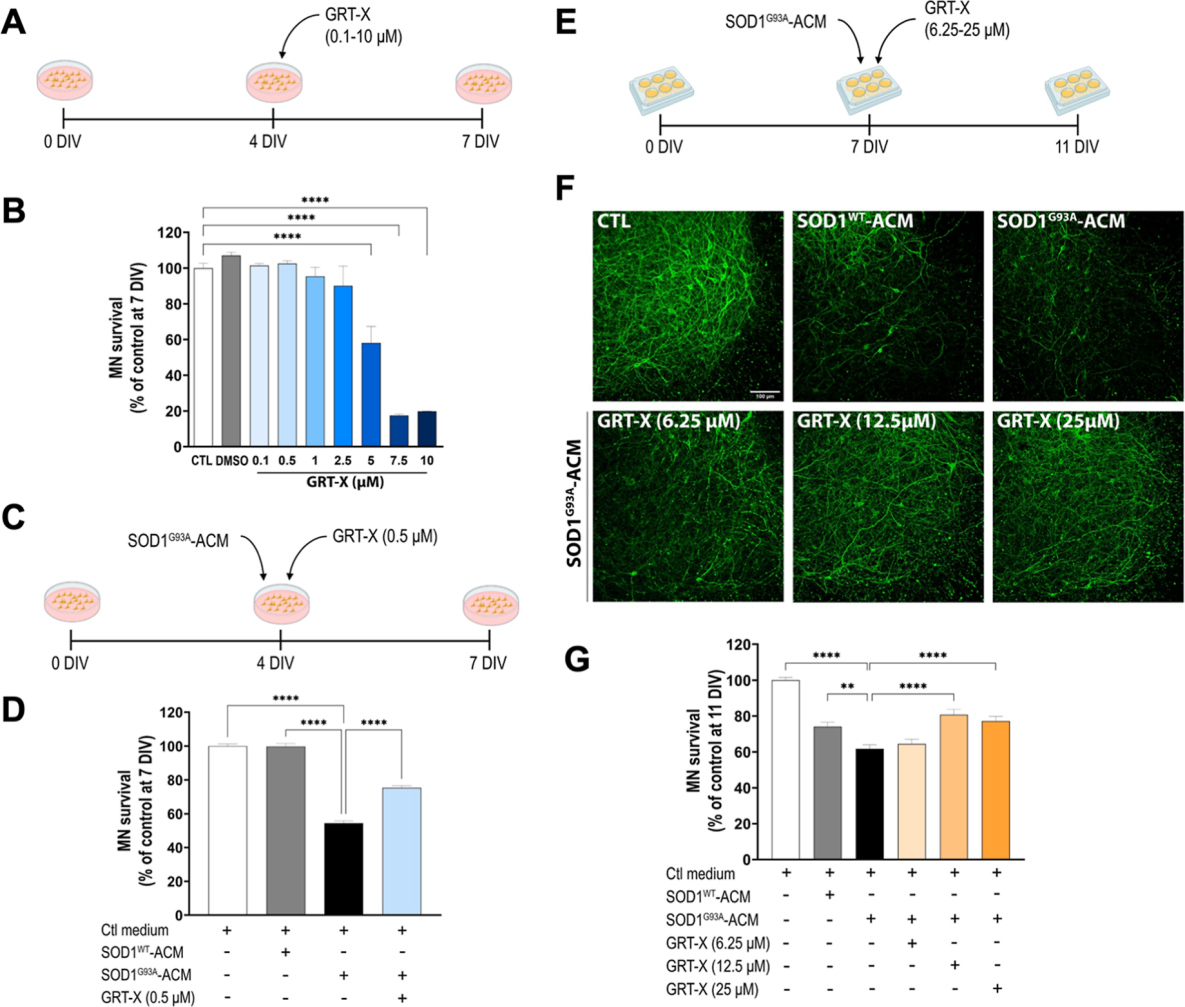
GRT-X treatment rescues MNs from cell death induced by mouse SOD1^G93A^-ACM (astrocyte-conditioned media). (A) Experimental design of GRT-X toxicity assessment in spinal MN cultures derived from WT rats. (B) Graph showing the percentage of MN survival (SMI32^+^/MAP2^+^ cells) in rat primary spinal cord cultures (VSCNs) exposed to increasing concentrations of GRT-X from 0.1 to 10 μM. Untreated (CTL) and vehicle (DMSO) were used as controls. (C) Experimental design to test the effects of GRT-X treatment on VSCNs exposed to mouse SOD1^G93A–^ACM. (D) Bar graph showing that GRT-X (0.5 μM) rescues MNs in VSCNs treated with SOD1^G93A^-ACM. (E) Experimental design to test the effects of GRT-X treatment on SCOC exposed to SOD1^G93A^-ACM. (F) Representative images of SMI32^+^-MNs (green) in the ventral horn of spinal cords at 11 DIV under the tested conditions. Scale bar 100 μm. (G) Bar graph showing that GRT-X at 12.5 and 25 μM rescues MNs in SCOCs treated with SOD1^G93A^-ACM. Data are shown as mean ± SEM. One-way ANOVA with Bonferroni’s post hoc test: ****p < 0.0001 and **p < 0.01 versus SOD1^G93A^-ACM condition and control medium.

To explore the beneficial effects of GRT-X in ALS in vitro models wild-type MNs were exposed to ALS-ACM harvested from primary astrocyte cultures that were generated from mice overexpressing the human SOD1^WT^ (control) and human SOD1^G93A^ protein. Immunofluorescence assays for Aldh1L1, S100β, EAAT2/GLT-1, and GFAP were performed to confirm that an efficient mature astrocyte phenotype was achieved for both control and ALS primary mouse astrocytes (Supporting Information, Figure 2). In VSCNs, these ACMs were added at a 1:8 dilution at 4 DIV for 3 days (Figure 3C). The dilutions of both the control and mutant SOD1 ACMs were based on the maximum concentration of SOD1^WT^-ACM that did not induce significant MN death in the VSCNs. The same dilutions were used in the experiments with SCOCs (see below). In agreement with previous studies,^13,15,16^ exposure of the VSCNs to SOD1^G93A^-ACM, but not SOD1^WT^-ACM, induced 46% of MN death (54 ± 1% MN preserved) (Figure 3D). We found that the addition of GRT-X in a concentration of 0.5 μM to these cultures prevented MN death, with MN survival rates of 75 ± 1%. In SCOCs, these ACMs were added at 7DIV for 4 days (Figure 3E). Addition of SOD1^G93A^-ACM to SCOCs induced MN loss, around 40% (62 ± 2% MN preserved), reaching a significant difference between control and SOD1^WT^-ACM (Figure 3F,G). When GRT-X was added simultaneously with the ACMs to the SCOCs, the 6.25 μM concentration was ineffective, while higher GRT-X concentrations of 12.5 and 25 μM significantly preserved MN viability (81 ± 3; 77 ± 3% MN preserved, respectively) (Figure 3F,G). SOD1^WT^-ACM led to a significant MN loss of around 25% (74 ± 2% MN preserved) compared to the control condition. The toxic effect of SOD1^WT^-ACM might be explained by previous studies in which expression of human SOD1^WT^ in aged mice triggered astrocytic reactivity and MN loss in the mouse spinal cord.^49^

In agreement with previous studies, we found that mouse SOD1^G93A^-ACM^13,16,18^ induce robust MN cell death in healthy spinal cord cultures. Interestingly, here we show for the first time that these human and mouse ALS/FTD-ACMs also cause the death of MNs in organotypic spinal cord cultures. SCOC is a more complex in vitro model to evaluate the neuroprotective effects on MNs since the anatomical organization of the neural circuitry and neuronal/non-neuronal cellular stoichiometry are preserved in the cultured spinal cord. These characteristics make SCOCs a good model to test the effects of new candidate compounds that may be studied from an integrated perspective.^50,51^

### GRT-X Treatment Preserves MN Survival in VSCNs and SCOCs Exposed to Human SOD1^D90A^-ACM

We also wanted to determine the beneficial effects of GRT-X using human ALS-ACM. For this, we generated mature astrocytes from an ALS patient’s iPSCs carrying the D90A mutation in SOD1 (SOD1^D90A^) and from control subject iPSCs (SOD1^WT^); we used similar protocols as recently described for the generation of TDP43 and control subject mature astrocytes.^11^ Immunofluorescence assays for CD44, Cx43, and S100β were performed to confirm that an efficient mature astrocyte phenotype was achieved for both control and ALS patient-derived iPSCs (Supporting Information, Figure 3). Comparing control human iPSC-derived astrocytes with control mouse primary spinal cord astrocytes further revealed that both cell types exhibit comparable expression levels of mature astrocyte markers, including ALDH1L1, S100β, EAAT2/GLT-1, and Cx43 (Supporting Information, Figure 4). However, and as expected, GFAP is markedly more prominent in primary mouse astrocytes, reflecting a reactive state induced by the tissue dissection from neonatal spinal cords and subsequent culture under serum-containing conditions (see ref 10 and references herein).

Next, we collected conditioned medium from human ALS astrocytes and assessed the toxicity of SOD1^D90 V^-ACM (1:6 dilution) to MNs in VSCNs (Figure 4A,B). The dilutions of both the control and mutant SOD1 ACMs were based on the maximum concentration of SOD1^WT^-ACM that did not induce significant MN death in VSCNs. The same dilutions were used in the experiments with SCOCs (see below). Controls included SOD^WT^-ACM and the control medium. In agreement with previous studies studying human iPSC- or iNPC-derived astrocytes ACM carrying mutations in SOD1,^20,21^ we found that SOD1^D90 V^-ACM, but not controls, led to a robust 40% MN death (59 ± 6% MN preserved) (Figure 4B). Next, two different concentrations of GRT-X, 1 and 2.5 μM, were tested to evaluate its neuroprotective capacity against SOD1^D90A^-ACM. Results showed a concentration-dependent rescue capacity of GRT-X, being statistically significant at both concentrations and resulting in a full rescue at the highest GRT-X concentration (89 ± 2; 104 ± 5% MN preserved) (Figure 4B).

**Figure 4:**
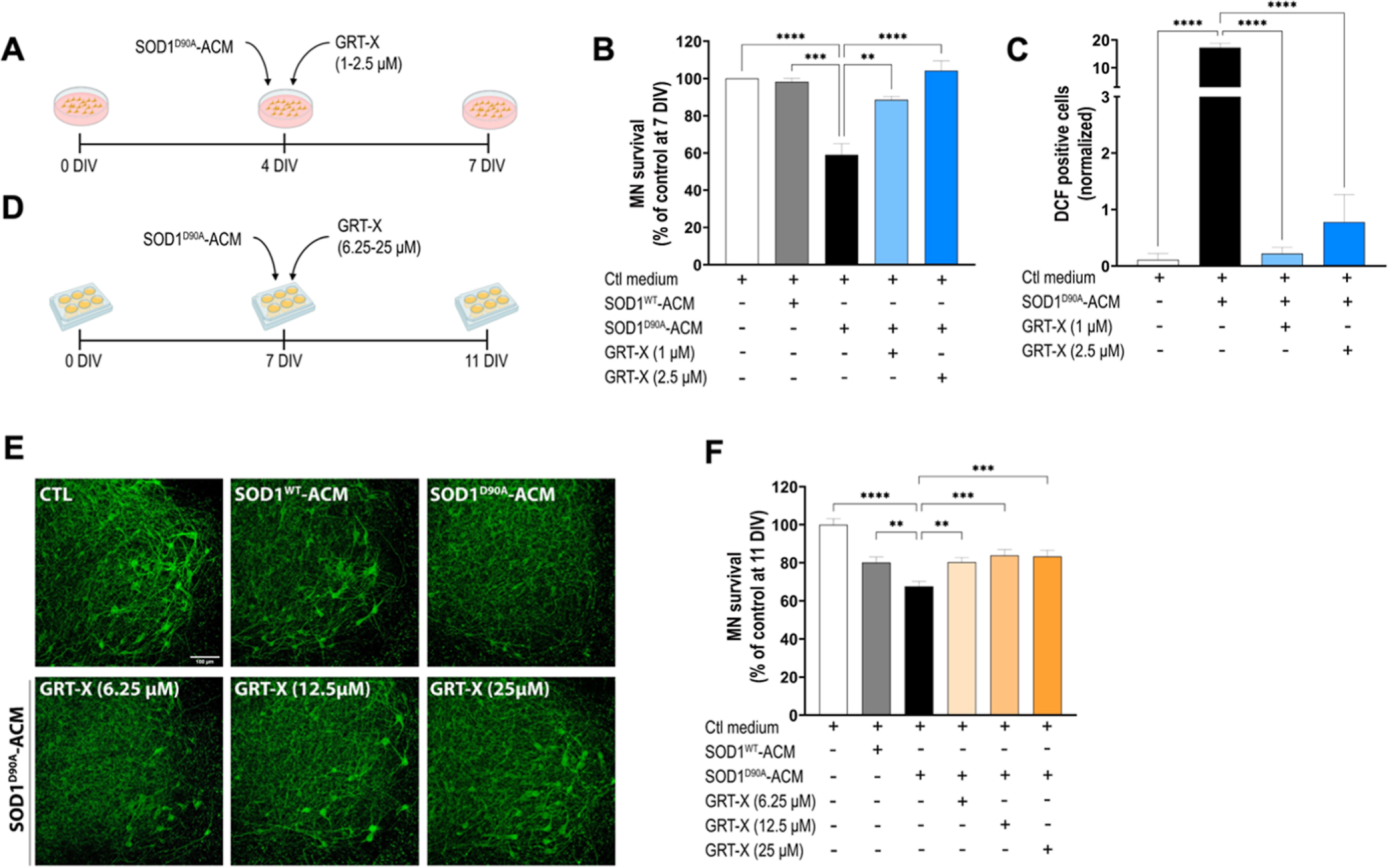
GRT-X treatment rescues MNs from cell death induced by human SOD1^D90A^-ACM (astrocyte-conditioned media). (A) Experimental design to test the effects of GRT-X treatment on rat primary spinal cord cultures (VSCNs) exposed to human SOD1^D90A^-ACM (diluted 1:6). (B) Bar graph showing that GRT-X rescues MNs in VSCNs treated with human SOD1^D90A^-ACM. (C) Quantification of the intracellular ROS/RNS levels in VSCNs by measurement of DCF (CM-H_2_DCF-DA) fluorescent neurons shows that GRT-X reduces ROS generation. (D) Experimental design to test beneficial effects of GRT-X treatment on SCOCs exposed to human SOD1^D90A^-ACM. (E) Representative images of SMI32^+^-MNs (green) in the ventral horn of spinal cord hemislices at 11 DIV under the tested conditions. Scale bar 100 μm. (F) Bar graph showing that GRT-X at all tested concentrations rescues MNs from cell death induced by human SOD1^D90A^-ACM. Data are mean ± SEM. One-way ANOVA with Bonferroni’s post hoc test: ****p < 0.0001; ***p < 0.001; **p < 0.01 versus human SOD1^D90A–^ACM condition and control medium.

Increased ROS/RNS levels are a shared feature of ALS patients and in vitro and in vivo ALS models. As previously demonstrated, mouse SOD1^G93A^-ACM, SOD1^G86R^-ACM, and TDP43^A315T^-ACM led to rapid increases in intracellular ROS/RNS levels in VSCNs, measured with DCF.^15,16^ CM-H_2_DCF-DA is a nonfluorescent dye that is hydrolyzed intracellularly and the oxidation of the DCFH group results in the formation of the fluorescent product DCF. Therefore, increases in fluorescent DCF intensities denote increased intracellular ROS/RNS levels. While under control conditions very few neurons exhibited detectable DCF levels, the application of SOD1^D90A^-ACM strongly increased neuronal DCF levels (17 ± 2 DCF positive cells). Application of both test concentrations of GRT-X, 1 and 2.5 μM, markedly reduced DCF counts to essentially control levels (0.2 ± 0.1; 0.8 ± 0.4 DCF positive cells, respectively) (Figure 4C).

In SCOCs, MN survival was assessed at 11 DIV after 4 days of chronic exposure to human SOD1^D90A^-ACM, SOD1^WT^-ACM, or control ACM (Figure 4D–F). The exposure to SOD1^D90A^-ACM induced a significantly stronger reduction of 32% (68 ± 3% MN preserved) in the number of SMI-32-labeled MNs in the ventral horn. The three concentrations of GRT-X tested (6.25, 12.5, and 25 μM) significantly rescued MN death induced by SOD1^D90A^-ACM (80 ± 2; 84 ± 3; 83 ± 3% MN preserved, respectively) (Figure 4E,F). The presence of human SOD1^WT^-ACM in SCOCs caused a significant MN death of around 20% (80 ± 3% MN preserved) compared to control slices, similarly to the effect observed after mouse SOD1^WT^-ACM addition.

GRT-X preserves MNs from human SOD1-ACM^20,21^ in both VSCNs and SCOCs. Similar to our previous findings reported here, the neuroprotective concentrations of GRT-X in primary VSCNs were lower than those required in SCOCs. While the reasons for this observation are not known, an explanation may be that Kv7.2/3 channel activation could be more effective in preserving MNs in primary VSCNs. Similarly, cultures of ALS patient-derived MNs may be more responsive to Kv7.2/3 channel activation, considering previous studies with retigabine, an approved antiepileptic drug also tested in a Phase II clinical trial in people with ALS.^41^ Retigabine activates Kv7.2/3 and hyperpolarizes the resting membrane potential,^40^ although with lower potency and efficacy than GRT-X.^23^ In cultures of human ALS patient-derived MNs, retigabine in concentrations of 1 and 10 μM hyperpolarized and thus stabilized the membrane resting potential, blocked disease hyperexcitability, and improved survival of MNs.^40^ Additionally, human SOD1-ACM^WT^ is also detrimental to SCOCs, potentially due to the presence of astrocytes in this culture model, as noted previously. We have also found that GRT-X reduces ROS/RNS levels in VSCNs when exposed to SOD1^D90A^-ACM. Previous studies using TSPO ligands have shown reduced ROS production in different cell types, such as isolated cardiomyocytes^52^ and endothelial cells.^53,54^ While the exact mechanism has not been elucidated, it was demonstrated that treatment with TSPO ligands in endothelial cells decreased (or reverted increased) ROS production and increased catalase activity and glutathione levels.^53^ Therefore, we hypothesize that GRT-X acts similarly to other TSPO ligands in reducing the ROS/RNS levels in our experiments. TSPO function has also been linked to mitochondrial energetic metabolism. The treatment with TSPO agonists Ro5–4864 and PK11195 had a stimulatory effect on basal respiration and ATP-related respiration in BV2 microglial cells.^55^ In addition to, TSPO KO reduces mitochondrial membrane potential, impairment of mitochondrial function, and inhibition of oxidative phosphorylation in BV2 microglial cells.^56^ Thus, activation of TSPO by GRT-X could contribute to maintaining mitochondrial energetic metabolism and, consequently, participate in MN preservation.

### GRT-X Treatment Preserves the Number of MNs in VSCNs and SCOCs Exposed to Human TDP43^A90 V^-ACM

We also evaluated the toxicity of ACM (1:6) collected from human iPSC-derived astrocytes generated from an ALS/FTD patient carrying an A90 V mutation in TARDBP (TDP43^A90 V^) and a healthy family member (TDP43^WT^).^12,13,46^ In agreement with our previous study,^12,13^ chronic exposure of VSCNs for 4 days to TDP43^A90 V^-ACM, unlike TDP43^WT^-ACM, induced around 50% of MN death (48 ± 5% MN preserved). The dilutions of both the control and mutant TDP43 ACMs were based on the maximum concentration of TDP43^WT^-ACM that did not induce a significant MN death in VSCNs. The same dilutions were used in the experiments with SCOCs (see below). In VSCNs, we found that both test concentrations of GRT-X, 1 and 2.5 μM, effectively reduced the TDP43^A90 V^-ACM induced MN death (85 ± 1% and 84 ± 1% MN preserved, respectively) (Figure 5A,B).

**Figure 5:**
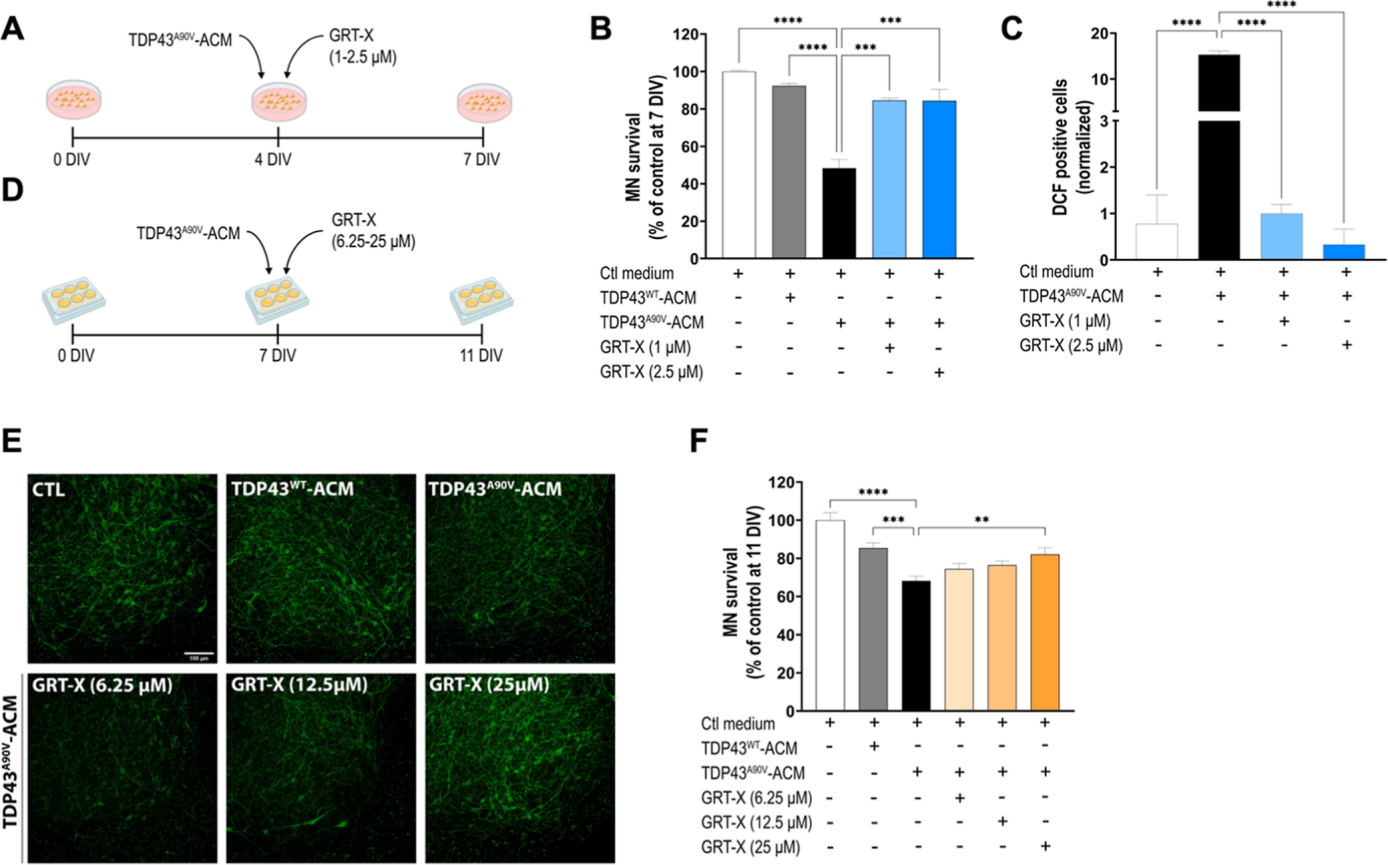
GRT-X treatment rescues MNs from cell death induced by human TDP43^A90 V^-ACM (astrocyte-conditioned media). (A) Experimental design to test the effects of GRT-X treatment on rat primary spinal cord cultures (VSCNs) exposed to human TDP43^A90 V^-ACM (diluted 1:6). (B) Bar graph showing that GRT-X preserves MN survival in VSCNs treated with human TDP43^A90 V^-ACM. (C) Quantification of the intracellular ROS/RNS levels in VSCNs by measurement of DCF (CM-H_2_DCF-DA) fluorescent neurons, showing that GRT-X reduces ROS generation. H_2_O_2_ (200 μM for 20 min) served as a positive control and to normalize the number of DCF-positive cells after ACM application. (D) Experimental design to test the effects of GRT-X treatment on SCOCs exposed to human TDP43^A90 V^-ACM. (E) Representative images of SMI32^+^-MNs (green) in the ventral horn of spinal cord hemislices at 11 DIV under the tested conditions. Scale bar 100 μm. (F) Bar graph showing that 25 mM GRT-X rescues MNs from cell death induced by TDP43^A90 V^-ACM in SCOCs. Data are mean ± SEM. One-way ANOVA followed with Bonferroni’s post hoc test: ****p < 0.0001, ***p < 0.001; **p < 0.01 versus TDP43^A90 V^-ACM condition and control medium.

Similar to SOD1^D90A^-ACM, the application of human TDP43^A90 V^-ACM led to an increase in DCF levels (15.3 ± 0.7 DCF positive cells) compared to the control medium (0.8 ± 0.6 DCF positive cells). We found that the addition of GRT-X, at 1 μM and especially at 2.5 μM, strongly reduced DCF levels (0.9 ± 0.2; 0.3 ± 0.3 DCF positive cells, respectively), leading to DCF levels found under the control condition (Figure 5C).

In SCOCs, exposure of TDP43^A90 V^-ACM caused MN death (68 ± 2% MN preserved) compared to TDP43^WT^-ACM and control conditions (Figure 5D–F). While GRT-X at 6.25 and 12.5 μM did not cause significant MN preservation (74 ± 3; 76 ± 2% MN preserved) compared to ACM-TDP43^A90 V^ alone, treatment with 25 μM GRT-X increased survival of MNs (85 ± 3% MN preserved) and reverted toxicity induced by human TDP43^A90 V^-ACM. As for previous experiments with ACMs in SCOCs, TDP43^WT^-ACM induced mid cell death (85 ± 3% MN preserved) compared to control medium, as shown in a previous study.^57^

As with other conditioned media in this study, human TDP43-ACM is harmful to VSCN, as previously documented;^12,13^ however, for the first time, we also demonstrate its detrimental effects on SCOCs. Consistent with the findings throughout this study, GRT-X displays neuroprotective properties in both VSCNs and SCOCs. Additionally, GRT-X significantly reduces ROS/RNS levels, which may partially explain its neuroprotective mechanism. These findings underscore GRT-X’s potential to counteract SOD1 and TDP43-induced toxicity, reinforcing its value as a neuroprotective agent in ALS.

Key findings of our preclinical proof-of-principle study are that GRT-X consistently protected MNs in a series of in vitro experiments. Rat MNs exposed to glutamate or to human or mouse ALS/FTD-ACM showed significantly higher survival rates and reduced ROS/RNS levels after treatment with GRT-X. Our data suggest that the decrease in hyperexcitability, reduction of ROS/RNS levels, maintenance of mitochondrial energetic metabolism and homeostasis, and stimulation of neurosteroidogenesis contribute to this neuroprotective effect. Nevertheless, additional studies are required to prove which molecular mechanisms and pathways underlie the observed effects produced by the dual Kv7.2/3 channel/TSPO receptor activation of GRT-X.

Another key outcome is that the GRT-X compound preserves MNs against toxic damage in vitro, similar to riluzole, the first treatment approved for ALS. While riluzole has a wide range of effects, at clinically relevant concentrations, this pharmacological agent preserves MNs mainly by reducing repetitive firing (inhibiting Na^+^ currents) and glutamatergic neurotransmitter release.^58,59^ Even though GRT-X differs in targets from the drug riluzole, the efficacy profile of GRT-X against ACM-induced toxicity in cultured MNs was similar to that found in previous studies on riluzole^15,16^ (see also Table 1). Both were able to significantly promote MN survival and restore ROS/RNS levels in primary MN spinal cord cultures exposed to ACM derived from astrocytes harvested from transgenic mice or from human astrocytes generated from patients’ iPSCs carrying ALS or FTD causing mutations.

**Table 1:** Comparison of Effects on ACM-Induced Toxicity in Cultured MNs by GRT-X and Riluzole, a Clinically Approved Treatment for ALS^a^

GRT-X (present data)			riluzole^14^		
ACM-type	tested concentrations: (μM)	treatment effects: ACM + GRT-Xversus ACM alone	ACM-type	tested concentration	treatment effects: ACM + riluzole versus ACM alone
mouse SOD1^G93A^-ACM^b^	0.5	MN survival rate: 75 ± 1% **** compared to 54 ± 1% ROS/RNS levels: n.t.	mouse SOD1^G93A^-ACM^b^	0.1 μM	MN survival rate: 91 ± 5% *** compared to 57 ± 9% ROS/RNS levels: 3 ± 1 *** compared to 72 ± 15
human SOD1^D90A^-ACM^c^	1/2.5 μM	MN survival rates: 89 ± 2% **/104 ± 5% **** compared to 59 ± 6% ROS/RNS levels: 0.2 ± 0.1 ****/0.8 ± 0.4 **** compared to 17 ± 2	n.t.	n.t.	n.t.
human TDP43^A90 V^-ACM^d^	1/2.5 μM	MN survival rates: 85 ± 1% ***/84 ± 6% *** compared to 48 ± 5% ROS/RNS levels: 0.9 ± 0.2 ****/0.3 ± 0.3 **** compared to 15.3 ± 0.7	mouse TDP43^A315T^-ACM^e^	0.1 μM	MN survival rate: 74 ± 6% ** compared to 52 ± 5% ROS/RNS levels: 4 ± 3 *** compared to 78 ± 11

a Primary spinal cord neuronal cultures were exposed to diverse ACMs, and MN survival and DCF positive cells indicative of ROS/RNS levels were determined in the presence of mutant ACM alone (ACM) and after treatment with mutant ACM plus GRT-X (ACM + GRT-X) or riluzole (ACM + riluzole).

b ACM collected from primary mouse ALS astrocytes carrying ALS-causing mutations of human SOD1^G93A^.

c ACM collected from human-induced pluripotent stem cell (iPSC)-derived astrocytes carrying ALS-causing mutations of SOD1 (SOD1^D90A^-ACM).

d ACM collected from human iPSC-derived astrocytes carrying ALS/FTD-causing mutations of TDP43^A90V^.

e ACM collected from primary mouse ALS astrocytes carrying ALS/FTD-causing mutations of human TDP43^A315^. Data are shown as mean ± SEM ****p < 0.0001, ***p < 0.001; **p < 0.01 versus glutamate or ACM condition, respectively. n.e.: no effect; n.d.: not determined.

GRT-X preserves MN survival in SCOCs exposed to acute excitotoxic damage and rescues MNs from cell death in primary VSCNs and SCOCs exposed to ACM derived from astrocytes harvested from transgenic mice carrying ALS-causing mutation SOD1^G93A^ and, importantly, from human astrocytes generated from patients iPSCs carrying pathogenic mutated SOD1^D90A^ or TDP43^A90 V^. Altogether, these data confirm our hypothesis that the dual mechanism of Kv7.2/3 channel/TSPO receptor activation could be protective in in vitro models of ALS/FTD.

In summary, our data suggest that this dual-mechanism Kv7.2/3 channel/TSPO receptor activation may present a new therapeutic option for the pharmacological treatment of MN degenerative pathologies. If this mechanism is clinically translatable, it may have the potential to improve treatment options for patients with ALS, FTD, and other diseases related to degeneration of MNs.

## Methods

### Ethics Approval Statement

All protocols involving rodents (including rat ventral spinal cord cultures and mouse astrocyte spinal cord cultures; see below) were carried out according to the NIH and ARRIVE guidelines and the European Communities Council Directive 2010/63/EU. Protocols were approved by the Ethical and Biosecurity Committees of Universidad Andres Bello and the Ethics Committee of Universitat Autnoma de Barcelona.

### Pharmacological Treatments

GRT-X (N-[(3-fluorophenyl)-methyl]-1-(2-methoxyethyl)-4-methyl-2-oxo-(7-trifluoromethyl)-1H-quinoline-3-carboxylic acid amide) was synthesized by Grünenthal GmbH (Aachen, Germany). For SCOCs, 10 mM stock solutions of GRT-X in dimethyl sulfoxide (DMSO) were diluted with SCOC medium. Olesoxime and ICA-27243 stock solutions in DMSO were also diluted in SCOC medium. For VSCNs, 10 mM stock solutions of GRT-X in DMSO were diluted with a VSCN medium.

### SCOCs

SCOCs were prepared as previously described.^50,60^ Briefly, P7–P8 Sprague–Dawley rats were deeply anesthetized with pentobarbital, and spinal cords were aseptically harvested and placed in ice-cold Gey’s Balanced Salt Solution (Sigma-Aldrich) containing glucose (6.4 mg/mL). Once meninges were removed, spinal cords were transversally cut into 350 μm thick slices using a McIlwainTissue Chopper (The Mickle Laboratory Engineering Co.). L4–L5 lumbar sections were transferred on Millicell-CM porous membranes (0.4 μm; Millipore, Burlington, MA, USA) into a six-well plate containing 1 mL of incubation medium: 50% minimal essential medium (MEM) (Sigma, Cat. #M5775), 25 mM Hepes (Sigma, Cat. #H4034–25G), 25% heat-inactivated Horse Serum (Gibco, Cat. #2605088), 2 mM glutamine (INC Biomedical Inc., Cat. #101806), and 25% Hank’s Balanced Salt Solution (HBSS) supplemented with 25.6 mg/mL glucose (Gibco, Cat. #14175-095). Cultures were left to stabilize for 7 DIV at 37 °C and 5% CO_2_, and thereafter, the medium was changed twice a week until stress induction.

### Olesoxime, ICA-27243, and GRT-X Treatment under Excitotoxic Stress in SCOCs

Excitotoxicity was induced at 15 DIV in SCOCs by transferring slices to Locke solution (137 mM NaCl, 2.5 mM CaCl_2_, 5 mM KCl, 5.6 mM d-glucose, 0.3 mM KH_2_PO_4_, 4 mM NaHCO_3_, 0.3 mM Na_2_HPO_4_, 0.01 mM glycine, and 10 mM Hepes; pH 7.2) containing l-glutamic acid (Sigma-Aldrich, cat. no. G8415) at a final concentration of 50 μM for 30 min. Following, olesoxime was administered at concentrations of 2.5 μM, 5 μM, 10 μM, and 20 μM, all dissolved in DMSO. The cultures were maintained until the 20 DIV. Combinational treatment with the unimodal compounds olesoxime and ICA-27243 consisted of the simultaneous addition of the same concentrations of both compounds 2.5 μM, 5 μM, and 10 μM each from 15DIV until 20DIV. For the treatment with the dual-mode of action compound, GRT-X at 6.25 μM, 12.5 μM, and 25 μM was added simultaneously to the excitotoxic solution. DMSO at the highest concentration used for GRT-X treatment was added as a vehicle condition. Locke solution without glutamate was also added to control and vehicle conditions without glutamate. After treatments, slices under different conditions were maintained for 5 DIV, then, fixed with 4% paraformaldehyde (PFA) and immunostained to determine MN survival as detailed below.

### ACM Preparation from Primary Mouse Astrocyte Cultures

Primary mouse astrocyte cultures and ACM were prepared as described previously.^13,15,16^ Hemizygous transgenic mice carrying mutant human SOD1G93A (high copy no.; B6SJL; cat. no. 002726) transgenes were obtained from Jackson Laboratories (Bar Harbor, Maine, USA). Briefly, cultures of astrocytes were prepared from P1–P2 transgenic mice expressing human SOD1^WT^, SOD1^G93A^, and nontransgenic littermates (controls). Cultures were maintained in DMEM (Hyclone, Cat. #SH30081.02) containing 10% FBS (Gibco, Cat. #16000–044), 1% l-glutamine (Gibco, Cat. #25030-081), and 1% penicillin–streptomycin (Gibco, Cat. #15140-122) at 37 °C and 5% CO_2_. After 3 weeks, astrocyte cultures reached confluence, and residual iba1^+^-microglia cells were removed overnight (7 h) using an orbital shaker (200 rpm in the incubator).

Next, astrocyte cultures were fixed with 4% paraformaldehyde and immunostained with Iba1 (Supporting Information, Figure 2A). In addition, astrocyte cultures were stained for Aldh1L1, S100β, EAAT2/GLT-1, GFAP, Cx43, and microtubule-associated protein 2 (MAP2) (Supporting Information, Figures 2 and 4). Details on these primary antibodies are described in Supporting Information, Table 1. Antibody binding was visualized with the appropriate fluorescent secondary antibodies, as described in Supporting Information, Table 2. Immunolabeled APCs or astrocytes were documented on an inverted Nikon Eclipse Ti–U microscope equipped with a SPOT Pursuit USB Camera CCD (14 bit), Epi-FL illuminator, mercury lamp, and Sutter Smart-Shutter with a Lambda SC controller. Cells were photographed by using a 40× objective.

For the generation of ACM, medium was replaced with neuronal growth medium: 70% MEM (Gibco, Cat. #11090-073), 25% neurobasal medium (Gibco, Cat- #21103-049), 1% N_2_ supplement (Gibco, Cat. #17502-048), 1% l-glutamine (Gibco, Cat. #2503-081), 1% penicillin–streptomycin (Gibco, Cat. #15140-122), 2% horse serum (Gibco, Cat. #15060-114), and 1% sodium pyruvate (Gibco, Cat. #11360-070), as previously described (11,13,14). ACM was collected after 7 days, supplemented with 4.5 mg/mL d-glucose (final concentration), and stored at −80 °C. The SOD1^WT^-ACM and SOD1^G93A^-ACM used to evaluate MN survival were diluted 8-fold in primary MN spinal cord cultures [MEM supplemented with neurobasal medium, N_2_ supplement, l-glutamine, penicillin–streptomycin, horse serum, and sodium pyruvate] and stored at −80 °C. The SOD1^WT^-ACM used to evaluate MN survival was diluted 8-fold in primary MN spinal cord cultures [MEM supplemented with neurobasal medium, N_2_ supplement, l-glutamine, penicillin–streptomycin, horse serum, and sodium pyruvate] and 9-fold in SCOC medium [MEM medium supplemented with HEPES, heat-inactivated horse serum, glutamine, and HBSS supplemented with glucose].

### ACM Preparation from Human iPSC-Induced Astrocytes Cultures

Human mutant SOD1 ^61^ and mutant TDP43 ^62^ astrocytes were generated from fully reprogrammed iPSC lines that were previously induced from skin fibroblasts biopsies by retroviral transduction using the four Yamanaka factors (OCT4, SOX2, KLF4, and cMYC). The SOD1^D90A^ iPSC line (commercially available from WiCell, WC034i) was generated from a 50 year old female ALS patient, and the control iPSC line was generated from an age-matched (50 year old) healthy female (WiCell, STAN140i-243C1). The TDP43^A90 V^ iPSC line was generated from an ALS/FTD 75 year old male patient carrying an A90 V mutation in TARDBP (TDP43^A90 V^) and from a healthy subject (56 year old female, termed control), a family member without mutations.^62^ Differentiation to neural progenitor cells (NPCs) and mature astrocytes was performed as described previously for mutant TDP43 and control subject iPSCs.^12,13^ The same protocol was used here for mutant SOD1 and control subject iPSCs. Briefly, iPSCs were maintained in feeder-free conditions using mTeSR1 medium (STEMCELL Technologies, Cat. # 85850). EBs were generated in EB differentiation medium [KnockOut DMEM/F12 media (Gibco, Cat. #12660-012) supplemented with 10% KnockOut serum replacement (Gibco, Cat. #10828-028), 1x GlutaMax (Gibco, Cat. #35050-061), 1x NEAA (Gibco, Cat. #11140-050), and 2-mercaptoethanol (Sigma-Aldrich, Cat. #M3148)] and maintained in suspension for 1 week. Rosette-shaped neuroepithelial cells were obtained after plating the EBs in plates coated with poly-l-ornithine (Sigma-Aldrich, Cat. #P4957) and laminin (Sigma-Aldrich, Cat. #L2020) and grown for 1 week in Neural Induction Medium [KnockOut DMEM/F12 supplemented with N2 (Gibco, Cat. #17502-048), NEAA, 2 mg/mL heparin (Sigma-Aldrich, Cat. #H3149), and 10 ng/mL bFGF (Gibco, Cat. #PHG0021)]. Rosettes were manually isolated under the microscope, replated in plates pretreated with Matrigel (Corning, Cat. #354277), and grown for one more week in Neural Expansion Medium [Neurobasal supplemented with Glutamax, NEAA, B-27 (Gibco, Cat. #17504-044), and bFGF]. Rosettes were disaggregated using Accutase Cell Detachment Solution (EMD Millipore, Cat. # SCR005) to generate a monolayer culture of NPCs. NPCs were differentiated to astrocyte precursor cells by culturing for 2 weeks in astrocyte precursor medium [KO DMEM/F12, 1x StemPro NSCs Supplement (Gibco, Cat. #A10508-01), 10 ng/mL Activin A (Gibco, Cat. #PHC9564), 10 ng/mL Heregulin 1b (R&D Systems, Cat. #377-HB-050), 200 ng/mL IGF1 (R&D System, Cat. #P291-G1-200), 20 ng/mL bFGF, 20 ng/mL EGF (Gibco, Cat. #PHG0311), and 1x GlutaMAX.^63^ Then, precursor cells were incubated for 2 additional weeks in astrocyte maturation and maintenance medium [DMEM/F12, B27, 10 ng/mL Heregulin, 5 ng/mL BMP2 (BioVision, Cat. #4577-50) and 2 ng/mL CNTF (R&D Systems, Cat. #257-NT-010)].^64^ Next, immunofluorescence assays were performed as previously described^12,13^ to confirm the development from NPCs (Nestin^+^) to APCs (CD44^+^) (Supporting Information, Figure S3) and then to a mature astrocytic phenotype (S100β^+^, ALDH1L1, EAAT2, GFAP, and Cx43) of these cultures (see Supporting Information, Figures S3 and S4) (see Supporting Information, Tables 1 and 2 for details on antibodies).

For the generation of ACM, medium of d28 human iPSC-induced astrocyte cultures was collected under media conditions identical to those used for mouse astrocyte cultures (see above). Thus, the media was replaced with Neuronal Growth Medium [MEM (Gibco, Cat. #11090-073) supplemented with 25% Neurobasal media (Gibco, Cat. #21103-049), 1% N2 supplement (Gibco, Cat. #17502-048), 1% l-glutamine (Gibco, Cat. #25030-081), 1% penicillin–streptomycin (Gibco, Cat. #15140-122), 2% horse serum (Gibco, Cat. #15060-114; lot 1517711), and 1% sodium pyruvate (Gibco, Cat. #11360-070)]. ACM was collected after 7 days, supplemented with 4.5 mg/mL d-glucose (final concentration) and stored at −80 °C. TDP43^WT^-ACM, TDP43^A90 V^-ACM, SOD1^WT^-ACM, and SOD1^D90A^-ACM used to evaluate MN survival were diluted 6-fold.

### Primary MN Spinal Cord Cultures

Pregnant Sprague–Dawley rats were anesthetized with CO_2_, and primary ventral spinal cultures (VSCNs) were prepared from E14 pups.^13,15,16^ Briefly, spinal cords were removed and placed into ice-cold HBSS (Gibco, Cat. #14185-052) with 50 μg/mL penicillin/streptomycin (Gibco, Cat. #15070-063). Using a small razor blade, the spinal cord’s dorsal part was removed from the ventral part. The ventral cord was grinded and enzymatically treated by incubating for 20 min at 37 °C in prewarmed HBSS containing 0.25% trypsin (Gibco, Cat. #15090-046). Cells were maintained in neuronal growth medium (see above) for 7–9 DIV at 37 °C under 5% CO_2_, and supplemented with 45 μg/mL E18 chick leg extract; medium was refreshed every 3 days. After 7 DIV cells were fixed for MN survival analysis.

### GRT-X Concentration–Response Curve

Concentration–response curves of GRT-X were performed in both VSCNs and SCOCs. In VSCNs, after 4 DIV, the MN cultures were treated at 0.1 μM, 0.5 μM, 1 μM, 2.5 μM, 5 μM, 7.5 μM, and 10 μM of GRT-X and DMSO as a vehicle (at the highest concentration used for GRT-X treatment) for 3 days. At 7 DIV, cultures were fixed, and survival analysis was performed as detailed in the immunofluorescence section.

In SCOCs, after 7 DIV, slices were subjected to 1 μM, 2.5 μM, 6.25 μM, 12.5 μM, and 25 μM of GRT-X and DMSO as vehicle control (used at the highest concentration used for GRT-X) was added for 4 days. Then, at 11 DIV, cultures were fixed, and MN survival analysis was performed as detailed in the immunofluorescence section below.

### GRT-X Treatment under ACM

VSCNs were exposed at 4 DIV to the mouse SOD1^WT^-ACM and SOD1^G93A^-ACM (both diluted 1:8) or to the human SOD1^WT^-ACM, SOD1^D90A^-ACM, TDP43^WT^-ACM, and TDP43^A90 V^-ACM (all diluted 1:6). For studies performed with mouse ACMs, GRT-X was used at 0.5 μM, and for the ones with human ACMs, pharmacological treatment with GRT-X was applied at concentrations of 1 and 2.5 μM. Culture medium without ACM was used as a control (CTL medium). At 7 DIV, cultures were fixed, and immunostaining analysis was performed to determine MN survival.

The SCOCs were exposed at 7 DIV to the same mouse ACM (1:9) or human ACM (1:6) used in VSCN cultures. GRT-X was applied at 6.25 μM, 12.5 μM, and 25 μM in SCOCs until 11 DIV. Culture medium without ACM was added as a control (CTL medium). Finally, immunofluorescence was performed for MN labeling.

### MN Viability Analysis by Immunofluorescence

In VSCNs, MNs, and interneurons were immunolabeled and counted as previously described.^13,15^ Briefly, cultures were fixed with 4% PFA at 7 DIV and incubated with primary antibody against microtubule-associated protein 2 (MAP2, 1:200; Invitrogen; Cat. #OSM00030W) to label interneurons and MN and with anti-neurofilament H nonphosphorylated (SMI-32, 1:1,000, Biolegend; Cat. #8071701), specifically expressed in MNs in culture, as these SMI-32/MAP2-identified MNs are also positive for ChAT.^15^

After incubation of the appropriate fluorescent secondary antibodies, MNs were visualized with epifluorescent illumination on an Olympus IX81 microscope (equipped with a Q-Imaging Micropublisher 3.3 Real-Time Viewing camera) using a 20× objective. MAP2- and SMI32-positive neurons were counted off-line using Fuji ImageJ. At least 12 randomly chosen fields (≥400 cells) were analyzed to calculate the percentage of SMI-32-positive MNs within the total number of MAP2-positive cells per condition. Each condition was replicated in 3–4 independent cultures.

In SCOCs, MNs were immunolabeled as previously described.^43^ Shortly, slices were fixed with 4% PFA, blocked with 5% normal horse serum (Biowest; cat. no. S0910500) and 0.3% Triton-X-100 in PBS (PBS-TX) and incubated with primary antibody against SMI-32 (1:250, Biolegend; cat. no. 801701) for 48 h. After incubation of secondary antibody Alexa Fluor^488^ donkey antimouse IgG (1:500, Invitrogen; Cat. #A21202) and DAPI (1:2000, Sigma; Cat. #D9564-10MG), Z-stack fluorescence images were captured using a ZEISS LSM 510 Meta confocal microscope. SMI-32+ MNs were selected according to the following criteria: localization in the lateral side of ventral horns and polygonal shape with clear dendrites. Blind counting of MNs SMI-32+ cells meeting these criteria was performed in each stack of the spinal cord hemisection using the Cell Counter tool from ImageJ software. Each hemislice was considered independent, and at least 12 hemislices were used in each condition.

### Intracellular ROS/RNS Measurement with CM-H_2_DCF-DA

Intracellular levels of ROS and RNS were measured in VSCNs with CM-H_2_DCF-DA (Invitrogen, Cat. #C6827), as previously described.^15,16^ CM-H_2_DCF-DA solution (5 mM) was prepared in DMSO and diluted in culture medium to a final concentration of 1 μM. To facilitate CM-H_2_DCF-DA membrane penetration, avoid hydrolysis, and maintain cell integrity, 0.004% Pluronic acid F-127 (Invitrogen, Cat. No. P-3000MP) was added to the culture medium.^65^

After the application of ACM in VSCNs, cells were washed to remove ACMs and exposed to CM-H_2_DCF-DA at 37 °C in the dark, labeling both MNs and interneurons. CM-H2DCF-DA-containing culture medium was removed, and cultures were suspended in fresh culture medium (500 μL final volume). Next, cells were immediately imaged using an inverted Nikon Eclipse Ti–U microscope equipped with a SPOT Pursuit USB CameraCCD (14 bit), Epi-fl Illuminator, mercury lamp, and Sutter SmartShutter with a lambda SC controller. Exposure time was kept below 4 s with excitation and emission wavelengths 492–495 nm and 517–527 nm, respectively. At least three independent fields were used for each condition (all fields were exposed for the exact same amount of time), and at least 10 cells per field were used for the quantification of the fluorescence signal. Fluorescence intensity was calculated for each region of interest of the cell body using the image analysis module in ImageJ software. Cells with a relative intensity unit of ≥1.5 were counted as positive. In all experiments, and as previously performed,^15,16^ H_2_O_2_ (200 μM for 20 min) served as a positive control and to normalize the number of DCF-positive cells after ACM application.

### Data Analyses

All data are expressed as mean ± SEM. One-way ANOVA, followed by Bonferroni’s posthoc test or Tukey’s posthoc test, was used to detect significant changes. Differences were considered significant at *p < 0.05, **p < 0.01, ***p < 0.001, ****p < 0.0001. At least 3–4 independent cultures/conditions were analyzed for each experiment. Cohen’s d was used to evaluate the effect size of the observed combination effects after treatment with equimolar combinations of olesoxime and ICA-27243 in comparison to the expected additivity effects. Cohen’s d is defined as the difference between the means of two groups divided by the pooled standard deviation; Cohen’s d values in the range of 0.2, 0.5, or ≥0.8 are interpreted as indicating small, medium, or large effect sizes, respectively.^66^

## Supplementary Material



## Data Availability

The data that support the findings of this study are available from the corresponding author upon reasonable request.

## Notes

Identification of MNs and interneurons in WT primary ventral spinal cord cultures; characterization of astrocytes and microglial cells in primary spinal cord cultures derived from control and SOD1^G93A^ transgenic mice; differentiation and characterization of iPSC-derived astrocytes from a control subject and an ALS patient harboring mutant SOD1^D90A^; characterization of control human iPSC-derived astrocytes and control mouse primary spinal cord astrocytes; primary antibodies used to characterize human and mouse astrocytes by immunostaining assays; and secondary antibodies used for the immunostaining assays (PDF)

## ¶

BlomsFunkeResearch GmbH, Gerhart-Hauptmann-Straße 36, D-52146 Würselen, Germany

## Abbreviations

ACM: astrocyte-conditioned medium
ALS: amyotrophic lateral sclerosis
FTD: frontotemporal dementia
GRT-X: N-[(3-fluorophenyl)-methyl]-1-(2-methoxyethyl)-4-methyl-2-oxo-(7-trifluoromethyl)-1H-quinoline-3-carboxylic acid amide
iPSCs: induced pluripotent stem cells
Kv7.2/3: voltage-gated potassium channels 7.2/3
MN: motoneuron
SCOC: spinal cord organotypic culture
SOD1: superoxide dismutase 1
TDP43: 43 kDa TAR DNA-binding protein
TSPO: mitochondrial translocator protein
VSCN: primary spinal cord culture.
