# Supplementary material for: Novel Dual Mechanism GRT‑X Agonist Acting on Kv7 Potassium Channel/Translocator Protein Receptor Prevents Motoneuron Degeneration Following Exposure to Mouse and Human Amyotrophic Lateral Sclerosis/Frontotemporal Dementia Astrocyte-Conditioned Media
Source: ACS Chem Neurosci. 2025 Jul 17;16(15):2887–900. doi: 10.1021/acschemneuro.5c00197 (PMC12333588; doi:10.1021/acschemneuro.5c00197)
Supplement: Supplementary file 1 [file cn5c00197_si_001.pdf]

Supplementary information

Novel dual mechanism GRT-X agonist acting on Kv7 potassium channel/TSPO receptor prevents motoneuron degeneration following exposure to mouse and human ALS/FTD astrocyte-conditioned media

Vera M. Masegosa[1,2+], Elsa Fritz[3+], Daniela Corvalan[3], Fabiola Rojas[3], Polett Garcés[3], Xavier Navarro[1,2], Petra Bloms-Funke[4,a], Brigitte van Zundert[3,5,6*], Mireia Herrando-Grabulosa[1,2*]

[1]Department of Cell Biology, Physiology and Immunology, Institute of Neurosciences, Universitat Autònoma de Barcelona, 08193 Bellaterra, Spain
[2]Centro de Investigación Biomédica en Red (CIBER), Instituto de Salud Carlos III, 28029 Madrid, Spain
[3]Institute of Biomedical Sciences (ICB), Faculty of Medicine & Faculty of Life Sciences, Universidad Andres Bello, 8370035 Santiago, Chile
[4]Grünenthal GmbH, 52099 Aachen, Germany
[5]Department of Neurology, University of Massachusetts Chan Medical School (UMMS), Worcester, MA 01655, USA
[6]Millennium Nucleus of Neuroepigenetics and Plasticity (EpiNeuro), 8370035 Santiago, Chile
[a]present address: BlomsFunkeResearch GmbH, Gerhart-Hauptmann-Straße 36, D-52146 Würselen, Germany

Authors:

Vera M. Masegosa: vera.martin@uab.cat

Elsa Fritz: mielsita@gmail.com

Daniela Corvalan: dacorvalan@uc.cl

Fabiola Rojas: lablpn@gmail.com

Polett Garcés: polettgarces@gmail.com

Xavier Navarro: xavier.navarro@uab.cat

Petra Bloms-Funke: pbf@blomsfunkeresearch.com

Corresponding authors:

Brigitte van Zundert: bvanzundert@unab.cl
Mireia Herrando-Grabulosa: Mireia.Herrando@uab.cat

Novel dual mechanism GRT-X agonist acting on Kv7 potassium channel/TSPO receptor prevents motoneuron degeneration following exposure to mouse and human ALS/FTD astrocyte-conditioned media

S1

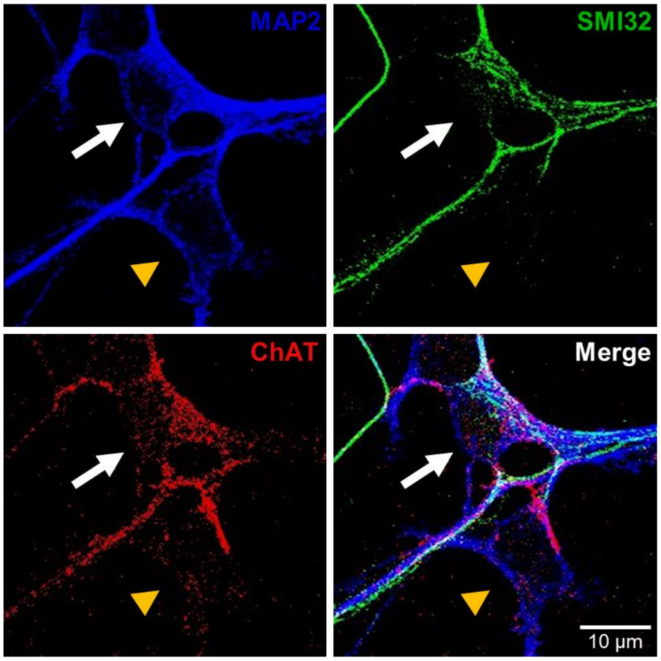

S1. Identification of motoneurons and interneurons in WT primary ventral spinal cord cultures. Confocal images showing immunostaining for MAP2 (blue), SMI32 (green), and ChAT (red) in rat ventral spinal cord cultures. $ChAT^{+}/SMI32^{+}/MAP2^{+}$ staining was used to identify motoneurons (white arrows), while $ChAT^{-}/SMI32^{-}/MAP2^{+}$ staining detected interneurons (yellow arrowheads).

Novel dual mechanism GRT-X agonist acting on Kv7 potassium channel/TSPO receptor prevents motoneuron degeneration following exposure to mouse and human ALS/FTD astrocyte-conditioned media

S2

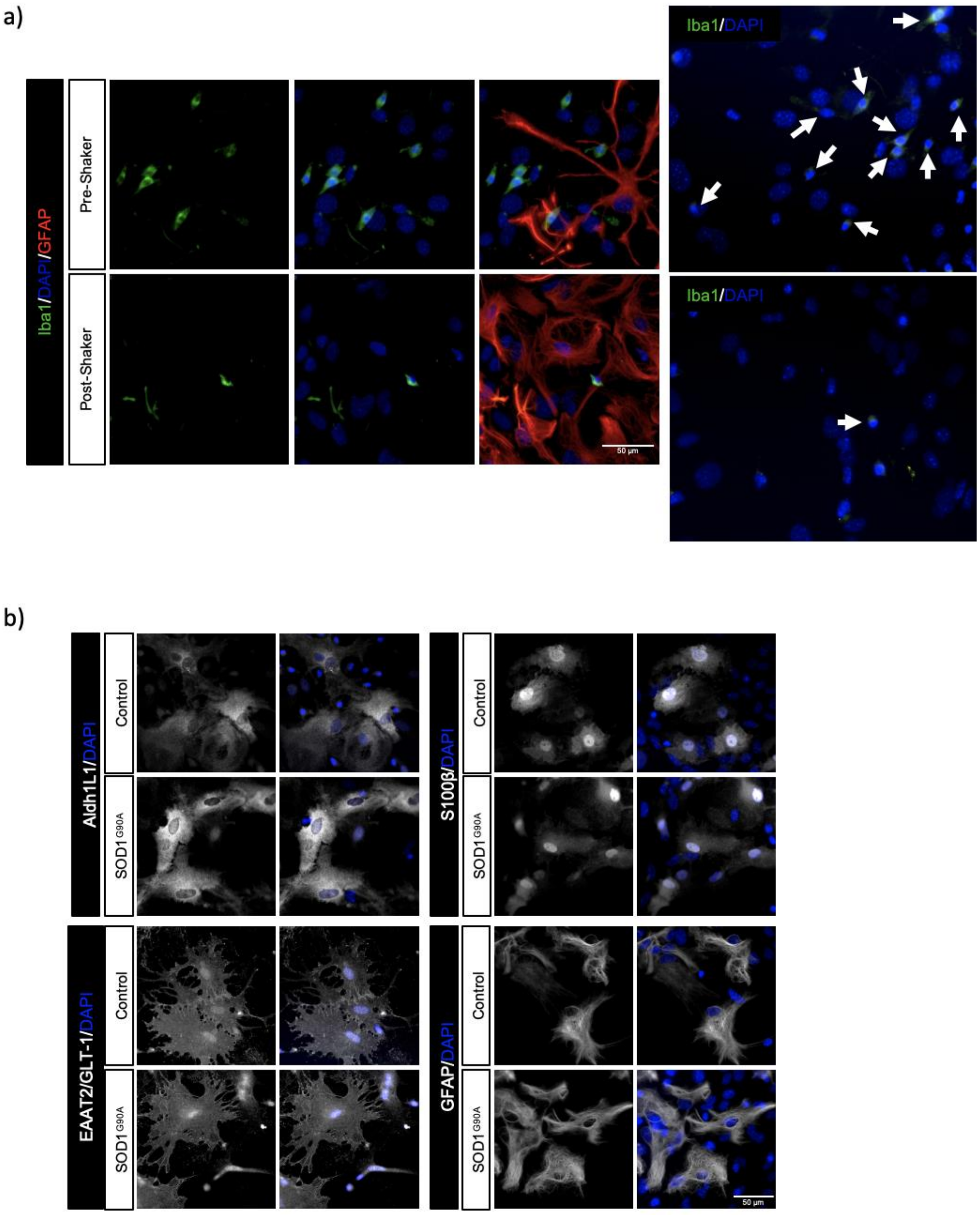

S2. Characterization of astrocytes and microglial cells in primary spinal cord cultures derived from control and SOD1^{G93A} transgenic mice. (a) Representative fluorescence microscopy images showing immunostaining for the microglial marker Iba1 (green) and astrocyte marker GFAP (red) prior (upper images) and after (lower images) performing overnight the shaker protocol. DAPI (blue) was used to visualize nuclei. Lower magnifications of Iba1/DAPI images are also shown (right). Before the shaking process, 9.0 % of the cells were Iba-positive (131 cells analyzed). After the shaking process, 1.6% Iba-positive cells were detected (270 cells analyzed). (b) Representative images showing control and SOD1^{G93A} astrocytes immunostained with the astrocyte markers (white) Aldh1L1, S100β, EAAT2 and GFAP (also indicating reactivity). DAPI (blue) was used to stain nuclei.

S3

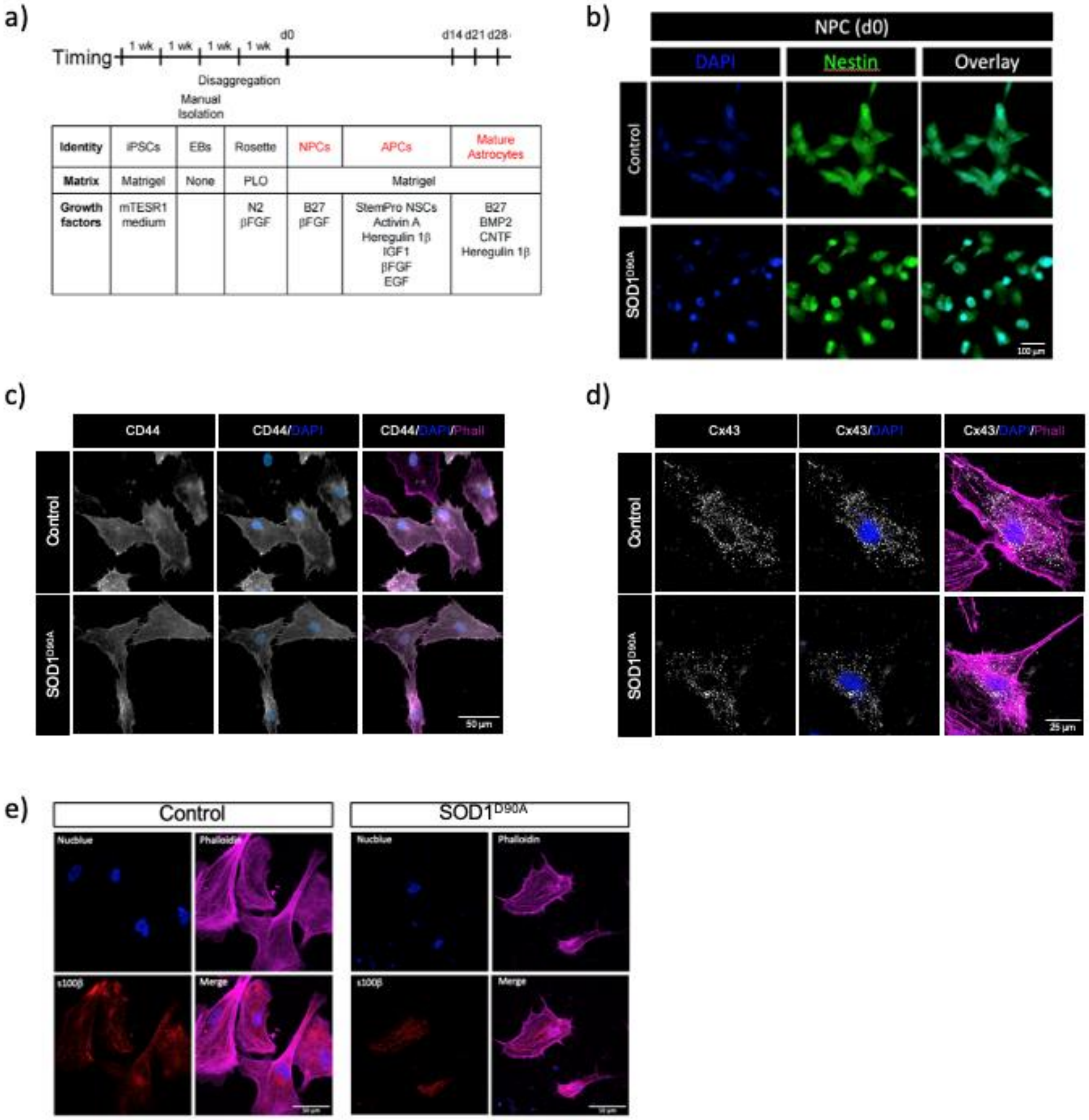

S3. Differentiation and characterization of iPSC-derived astrocytes from a control subject and an ALS patient harboring mutant SOD1^{D90A}. (a) Schematic protocol showing the stepwise differentiation process from human iPSCs to embryoid bodies (EBs), neural precursor cells (NPCs), astrocyte precursor cells (APCs), and finally mature astrocytes, with indication of duration in weeks (*wk*) and days (*d*). (b) Representative fluorescence microscopy images for NPCs (d0) derived from both a control subject and an ALS patient carrying the mutant SOD1^{D90A}, showing robust immunofluorescence staining for the NPC marker Nestin (green). Cell nuclei were visualized with DAPI (blue). (c-e) Immunofluorescence analysis performed on mature astrocytes (d28) derived from iPSCs of a control subject and an ALS patient. The images show the expression of the markers CD44 (c) and Cx43 (d) in white, along with phalloidin (magenta). Additionally, the mature astrocyte marker S100β is indicated in red (e). To stain nuclei (blue), DAPI or Nucblue were used as indicated.

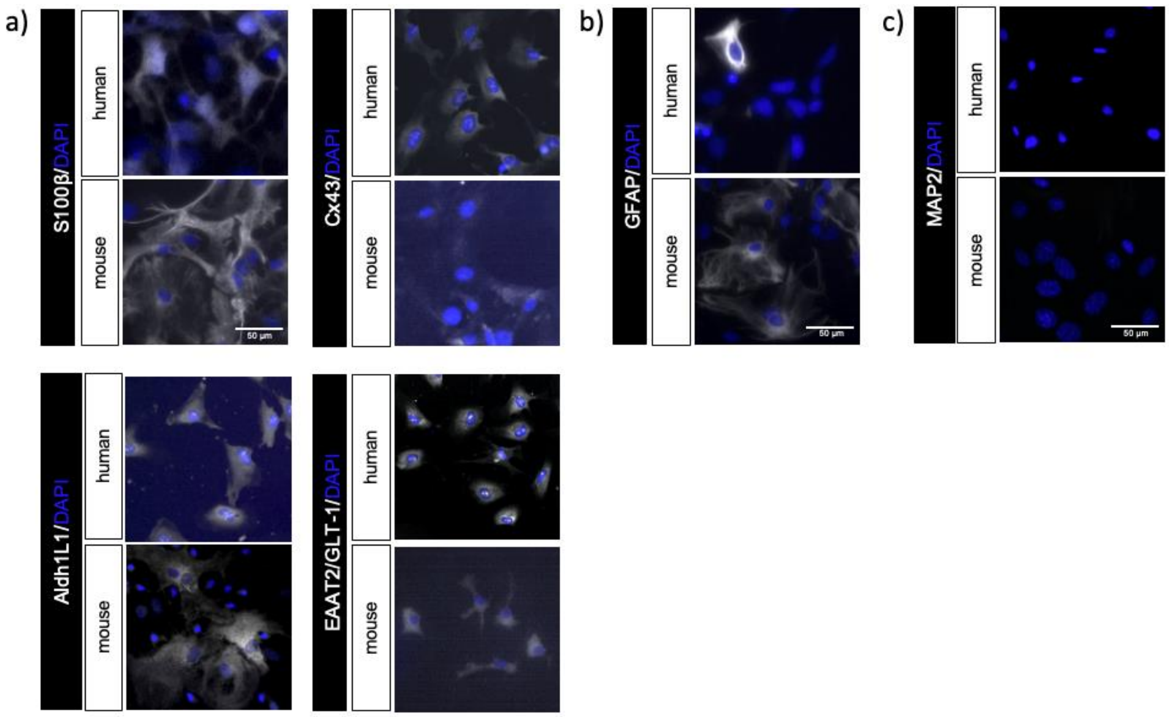

S4: Characterization of control human iPSC-derived astrocytes and control mouse primary spinal cord astrocytes. (a-c) Representative fluorescence microscopy images of control human iPSC-derived astrocytes (d28) and control mouse primary spinal cord astrocytes showing immunostaining for (a) the mature astrocyte markers S100β, ALDHL1, Cx43 and EAAT2/GLT-1, (b) GFAP, marker for reactive astrocytes, and (c) neuronal marker MAP2. To stain nuclei (blue), Nucblue was used as indicated.

Table S1: Primary antibodies used to characterize human and mouse astrocytes by immunostaining assays.

Primary antibody	Company	Catalog #	Host	Reacts to	Dilution
ALDH1L1	NeuroMab	75-140	Mouse monoclonal	Hum Ms Rat	1:50
ALDH1L1*	Abcam	ab87117	Rabbit polyclonal	Ms Rat	1:50
CD44	BD Biosciences	550392	Mouse monoclonal	Hum Ms	1:1000
Cx43	Invitrogen	13-8300	Mouse monoclonal	Hum Ms Rat	1:200
EAAT2/GLT-1	Invitrogen	PA5-17099	Rabbit polyclonal	Hum Ms Rat	1:50
EAAT2/GLT-1*	Sigma-Aldrich	AB1783	Guinea pig polyclonal	Hum Ms Rat	1:1000
GFAP	Dako	z0334	Rabbit polyclonal	Hum Ms Rat	1:1000
MAP2	Invitrogen	OSM00030W	Rabbit polyclonal	Ms Rat	1:600
Nestin	Millipore	MAB5326	Mouse monoclonal	Hum	1:500
S100β	Dako	Z0311	Rabbit polyclonal	Hum	1:1000
Iba1	Santa Cruz	sc-32725	Mouse monoclonal	Hum Ms Rat	1:150

*Antibody that was used for *Supplementary Figure 3*.

Table S2: Secondary antibodies used for the immunostaining assays.

Primary antibodie	Company	Catalog #	Host	Reacts to	Dilution
Alexa Fluor 488 Gt antiMs	Invitrogen	A-11029	Goat	Mouse (Ms)	1:500
Alexa Fluor 546 Gt antiMs	Invitrogen	A-11003	Goat	Mouse (Ms)	1:500
Alexa Fluor 633 Gt antiMs	Invitrogen	A-21052	Goat	Mouse (Ms)	1:500
Alexa Fluor 488 Gt antiRb	Invitrogen	A-11034	Goat	Rabbit (Rb)	1:500
Alexa Fluor 546 Gt antiRb	Invitrogen	A-10035	Goat	Rabbit (Rb)	1:500
